# A Balancing Act: How Professionals in the Foster Care System Balance the Harm of Intimate Partner Violence as Compared to the Harm of Child Removal

**DOI:** 10.1007/s42448-023-00153-0

**Published:** 2023-02-09

**Authors:** Laura Liévano-Karim, Taylor Thaxton, Cecilia Bobbitt, Nicole Yee, Mariam Khan, Todd Franke

**Affiliations:** 1 Luskin School of Public Affairs, UCLA, Los Angeles, CA USA; 2 School of Public Health, UC Berkeley, Berkeley, CA USA; 3 Charles R Drew/David Geffen School of Medicine, UCLA, Los Angeles, CA USA; 4 School of Law, UCLA, Los Angeles, CA USA; 5 UCLA Pritzker Center for Strengthening Children and Families, UCLA, Los Angeles, CA USA; 6 Fielding School of Public Health, UCLA, Los Angeles, CA USA; 7 David Geffen School of Medicine, UCLA, Los Angeles, CA USA; 8 Luskin School of Public Affairs, UCLA, Los Angeles, CA USA; 9 UCLA Pritzker Center for Strengthening Children and Families, UCLA, Los Angeles, CA USA

**Keywords:** Child welfare system, Intimate partner violence, Maltreatment

## Abstract

The striking prevalence of child exposure to intimate partner violence (IPV) and its associated adverse health outcomes necessitates a robust response from professionals who must grapple with the ethical dilemma of how to serve and support children in these circumstances. In 2020, 42 participants from four different professional backgrounds (attorneys, nonprofit leadership, licensed therapists, and social workers) were interviewed or participated in a focus group discussion. All groups acknowledged the shortfalls of current intervention practices, which often result in child removal. Group 1, which included social workers that work for children’s legal services, minor’s counsel, and Los Angeles Department of Child and Family Services social workers, were more conflicted in their recommendations for change. Some Group 1 participants recommended more training, while others thought more training would make little difference and recommended more substantial changes to prevent child removal when possible. Group 2, which included parents’ counsel, and Group 3, which included social workers, attorneys, and nonprofit leadership at IPV nonprofits, were more closely aligned in their recommendations, primarily focusing on systemic changes to the child welfare system. Participants whose employment required them to advocate for parents tend to view child removal from a non-offending parent as harmful for both the child and IPV survivor. These findings illuminate how the perspectives of these diverse participants are influenced by their professional and personal experiences.

## Introduction

In the USA, it is estimated that each year 15.5 million children are exposed to intimate partner violence (IPV) at home (Hamby et al., 2011). The lasting adverse effects of witnessing IPV during childhood have led some states to approach IPV as a form of child maltreatment. Los Angeles (LA) County is home to the largest child welfare system (CWS) in the country, with the Department of Children and Family Services (DCFS) caring for 29,458 children in March 2022 (Los Angeles County Department of Children & Family Services, 2022). While the available data does not specifically state how many of the children in Los Angeles’s foster care system have come under the supervision of DCFS as a result of witnessing IPV at home, from 2016 to 2019, between 10 and 18% of child abuse and neglect reports in California included allegations of IPV (Rebbe et al., 2021). The variability in the proportion of cases that included IPV is likely seasonal, with higher rates of IPV allegations occurring with school closures. Following the shutdowns and school closures during the COVID-19 pandemic in March 2020, the number of children in the CWS with cases involving IPV increased by 25% (Rebbe et al., 2021).

There is a well-documented association between IPV and child maltreatment. It has been suggested that neglect-related maltreatment is more likely to co-occur with IPV than abuse-related maltreatment (Rebbe et al., 2021). Multiple studies have found that in these cases, the co-occurring maltreatment likely results from a lack of appropriate supervision or emotional neglect (Dong et al., 2004; Henry, 2018; Victor et al., 2019). In 2010, a study utilizing survey data from across the USA found that 33.9% of youth who witnessed IPV experienced co-occurring maltreatment, with psychological abuse as the most frequently reported form of maltreatment at 23.4% (Hamby et al., 2010). Additionally, families experiencing IPV are more likely to have additional risk factors that can lead to child welfare involvement, such as a primary or secondary caregiver with substance abuse concerns (Kohl et al., 2005).

Even in isolation, violence occurring in the home is considered child neglect under several state laws that define abuse and neglect, including California’s Welfare and Institutions Code Sect. 300(b), known colloquially as a Failure to Protect law. As a result, parent-survivors, the parent harmed by the partner perpetrating violence, can become involved in the child welfare system and even dependency court when allegations are made against their abuser (Ogbonnaya & Pohle, 2013). In California, if a first responder finds that there is reasonable likelihood that IPV is present, known as a reasonable suspicion standard, a child welfare social worker will be assigned to the case to conduct an investigation (Los Angeles County Department of Children and Family Services, 2019a). This initial assessment allows the county to determine how the alleged violence impacts the child, what other risk factors may exist in the home environment, and what form of support or intervention would best serve the child and their family. Common interventions include anger management courses, parenting courses, counseling, or helping families access benefits they qualify for (Arango et al., 2014).

The social worker can keep the family under supervision, or if they determine the child is in immediate danger, they can remove them from home; in either situation, a case will be opened in Los Angeles Dependency Court (Welfare & Institutions Code, 1989; Los Angeles County Department of Children and Family Services, 2019a). Once a family enters dependency court, each actor is represented by an attorney (Lines, 2010). In Los Angeles County, DCFS is represented by County Counsel, the Children’s Law Center represents the child, and the Los Angeles Dependency Lawyers represent the parents. California has a right to counsel for all parents involved in a DCFS investigation. In 2021, Rebbe and colleagues (2021) found that for children under the age of five in California, 7.3% of IPV allegations led to a child being removed from the home. Placement with a relative outside of the home is more common; this occurs in 11% of cases, while 7% result in foster care (Kohl et al., 2005).

This study uses the term child welfare system (CWS) to refer to the broad ecosystem of government and nonprofit services that seek to care for children in the USA, including social workers (employed both by the government and by nonprofits), lawyers, and nonprofit leaders who operate in this child welfare ecosystem. All professionals’ legal and ethical responsibilities in the CWS, including lawyers and social workers, can be viewed as a balancing act. These professionals must always weigh the harm of exposure to witnessing IPV and foster care placement with the child’s best interests in mind.

Intimate partner violence (IPV) denotes harmful and complex circumstances involving power and control between intimate partners (United Nations (n.d.); Wang, 1996). IPV, not domestic violence, is used throughout this paper as our study focuses on children being exposed to violence between their parents or between one parent and their partner, not maltreatment directed towards the child. Specifically, this study examined abuse directed from one person to another, not situations where both members of the relationship are abusive towards each other. However, IPV can encompass situations where both members of the relationship are abusive towards each other. Domestic violence (DV) includes violence within intimate spaces, such as the home, instead of just intimate partnerships. It can also encompass harm to others residing within the intimate space, such as children and the elderly. The term “domestic violence” is more frequently used in the child welfare community; therefore, most of the participants in this study used this term during our interviews.

The striking prevalence of child exposure to IPV and its associated adverse health outcomes necessitates a robust response from professionals who must grapple with the ethical dilemma of best protecting children in these challenging circumstances. Research and advocacy efforts have indicated that child welfare practitioners lack the tools necessary to navigate and respond to IPV’s complexities (Fusco, 2013). There is additional complexity in addressing this issue as the impact of child removal and foster care placement in the context of IPV is not well understood. Therefore, this study explores the intersection of IPV and the CWS from the diverse perspectives of professionals who work with affected families. Furthermore, it intends to examine commonalities and differences between the perspectives of professionals working as part of the CWS ecosystem, grouping them based on the job duties and responsibilities.

## Background

### Risks and Consequences of Witnessing Intimate Partner Violence for Children

The trauma of IPV has profound health impacts on adult survivors and their children who witness this violence (Campbell, 2002; Campbell & Lewandowski, 1997). Witnessing IPV has been established as an adverse childhood experience (ACE), defined as severe, prolonged, or repetitive stress early in life without consistently nurturing adult relationships (Danese & McEwen, 2012). The relationship between ACEs and poor health has been tied to dysregulation of the hormonal stress response system, also known as the hypothalamic–pituitary–adrenal axis (Ridout et al., 2018). ACEs are linked to chronic activation of the stress response, resulting in significant morbidity and mortality risk over the life course (Bellis et al., 2015; Felitti et al., 1998; Fredland et al., 2008). A secure, consistent relationship between caregiver and child can help mitigate the toxic stress caused by ACEs (Danese & McEwen, 2012). However, IPV may interfere with a parent-survivor’s ability to provide optimal caregiving to their children due to state-imposed separation and violence-related mental health concerns, such as depression and anxiety (Mueller & Tronick, 2019).

Consequently, the parent-survivor may find it challenging to develop and maintain secure attachments with their child. They may be unable to buffer the harmful consequences of a child’s chronically activated stress response (Mueller & Tronick, 2019). In this way, IPV can overwhelm a child’s capacity for self-regulation and disrupt their developing sense of security, thereby elevating the risk for psychiatric disorders and poor cognitive development (Kessler et al., 2010; Mueller & Tronick, 2019).

### Consequences of Family Separation and Foster Care Placement for Children

Despite its goal to reduce harm and protect victims, the child welfare system (CWS) can produce intergenerational trauma in its separation of families (Fitzgerald et al., 2020). In cases of child maltreatment, foster care has been identified as a protective intervention. However, when a family is solely experiencing intimate partner violence (IPV), a situation that may be considered child neglect under many state laws, foster care placement may not be an appropriate response if alternative interventions could improve parenting capacity and child safety. Although the evidence base assessing the outcomes of child welfare involvement in situations where IPV is present is limited, there is some emerging quantitative and qualitative research to suggest that removing children from their families and placing them in foster care can lead to significant harm as well (Font & Gershoff, 2020; Raz & Sankaran, 2019).

Forced separation from a primary caregiver during childhood can be a significant adverse childhood experience (Choi et al., 2020). The trauma resulting from disrupted relationships between children and their primary caregivers may make it challenging for children in foster care to form secure attachments with other adults, including their new foster parents, increasing the risk of foster care placement disruption (Fisher et al., 2013). Children in foster care may also suffer the harmful effects of a chronically activated stress response in the absence of consistent and responsive caregiving due to further placement instability (Fisher et al., 2013).

The adverse health consequences of family separation and foster care placement are pervasive. Children forcibly separated from their primary caregivers are at increased risk for emotional and behavioral problems (Choi et al., 2020). They also face an increased risk of psychopathology in adulthood independent of child maltreatment (Naylor et al., 2019). Adults who experienced out-of-home foster placement between the ages of 2 to 4 were more likely to develop neurodevelopmental and psychiatric disorders than those who were not removed from their families as children but experienced similar life circumstances (Havlicek et al., 2013). Furthermore, two UK studies found that adults placed in out-of-home foster care as children saw an increase in all-cause mortality risk compared to those who experienced child maltreatment but remained with their families at home (Gao et al., 2017; Murray et al., 2020). For these reasons, the mental and physical health burden stemming from child welfare involvement, and more specifically foster care placement, cannot be underestimated when deciding to separate children from their families.

### Current Study 

This qualitative study explores the intersection of IPV and the child welfare system from the diverse perspectives of professionals who work with affected families. Using qualitative thematic analysis, this study explores how a person’s professional role as a parent or child advocate in the foster care system influences their perception of harm caused by witnessing IPV compared to the harm caused by system involvement, which may lead to family separation. Specifically, we seek to answer the following research questions:

- How do participants from different professional backgrounds consider the risk of harm to survivors of IPV and their child(ren) after child welfare system involvement in situations where the children may witness IPV?
- Do participants from different professional backgrounds perceive that there are alternative options beyond removing the child(ren) after child welfare involvement in situations where the children may witness IPV?

## Methods

This study analyzed pre-existing, self-collected qualitative data, as Heaton (2008) defined. Primary data collection was conducted via virtual Zoom interviews as part of a larger project by the UCLA Pritzker Center for Strengthening Children and Families examining the relationship between intimate partner violence (IPV) and Los Angeles County’s child welfare system (CWS). Data collection took place from August 2020 to December 2020. It involved semi-structured one-on-one interviews and focus group discussions (FGDs) in English. To recruit participants, researchers emailed supervisors at Los Angeles County’s Department of Children and Family Services (DCFS), supervisors at the Los Angeles Department of Mental Health, supervisors at legal services organizations that represent children in dependency court, supervisors at legal organizations supporting domestic violence survivors, and supervisors at domestic violence nonprofits. These emails asked the supervisors at these organizations whether they or any of their staff were interested in participating in a FGD for this study. At the end of each FGD, researchers asked whether the participant suggested we speak with anyone else. If a participant suggested someone else, researchers asked that participant to send an introductory email.

FGD composition was based only on availability, not professional background. Participants included social workers, nonprofit leadership, nurses, therapists, and attorneys working in the USA in various organizations such as nonprofits and community-based organizations, legal aid offices, and the Department of Children and Family Services (DCFS). In both one-on-one interviews and focus group discussions, participants were asked three questions:

1. Describe your role in your organization.
2. How do foster care and domestic violence intersect in your experience?
3. What needs to change for survivors of domestic violence and their children?

Facilitators would ask follow-up questions when appropriate, and participants could also ask questions or respond to another participant’s statements.

Primary data collection was completed with the approval of the UCLA Institutional Review Board (IRB). Results from this project have been published in the report by the UCLA Pritzker Center *Child Welfare and Domestic Violence: The Report on Intersection and Action,* published on May 12, 2021*.* Current, deidentified secondary data analyses were exempt from IRB revision by UCLA.

### Data Source

The qualitative data set used in this study included 21 transcriptions from over 13 h of audio recordings, 11 semi-structured one-to-one interview transcripts from almost seven hours of audio recordings (6:46:39), and ten focus group discussion transcripts of approximately six and a half hours of audio recordings (6:22:45). Overall, there were 42 participants. FGD size ranged from two to six participants, with an average of approximately three participants per focus group discussion. The majority of the participants were lawyers (19), followed by social workers (11), and members of nonprofit leadership (9).

### Data Analysis

As Heaton (2008) defined, researchers conducted a supra-analysis where the aims and focus of this secondary data analysis differed from the main purpose of the primary data collection. Researchers re-grouped participants by job within the child welfare system (CWS). This categorization resulted in three groups: Group 1, Group 2, and Group 3. The rationale for this initial categorization was the need to identify how to compare professionals’ perspectives given their current roles in Los Angeles County’s CWS.

Group 1 includes social workers that work for children’s legal services, minor’s counsel, and DCFS social workers. These professionals have a legal mandate to focus their work towards “the child’s best interest” (California Welfare & Institutions Code 1991). Yet, it is important to note that others have highlighted, and this research supported the view, that this legal duty to work in the child’s best interest is complicated through an individual’s conflicting duties and responsibilities to the state, the court, the child, and their personal moral beliefs (Berrick, 2019).

Group 2 participants are parents’ attorneys in the child welfare system. As the legal representatives of parents, including parent-perpetrators and parent-survivors in IPV situations, their job is to represent the interests of parents.

Group 3 participants included the leadership, attorneys, and social workers at domestic violence shelters or IPV nonprofits. Unlike Group 2 attorneys, Group 3 attorneys only represent the non-offending parent-survivor of IPV.

Each participant was assigned to a group. This process resulted in 28 Group 1 participants (five who participated in an interview and 23 in a FGD), six Group 2 (three who participated in an interview and three in a FGD), and eight Group 3 participants (three who participated in an interview and five in a FGD) (Table 1). Following the grouping of the participants, transcriptions were uploaded to the software Dedoose for qualitative coding.

**Table 1:** Participant demographics

Participant number	Profession	Education	Group number
1	Lawyer	JD	2
2	Social worker	MSW	1
3	Social worker	PsyD	3
4	Nonprofit leadership	BA	3
5	Lawyer	JD	1
6	Lawyer	JD	1
7	Social worker	BA	1
8	Social worker	MSW	1
9	Lawyer	JD	1
10	Lawyer	JD	1
11	Lawyer	JD	1
12	Lawyer	JD	1
13	Lawyer	JD	1
14	Lawyer	JD	1
15	Lawyer	JD	1
16	Social worker	AA	1
17	Social worker	MSW	1
18	Lawyer	JD	2
19	Lawyer	JD	2
20	Social worker	MSW	1
21	Nonprofit leadership	MS	1
22	Social worker	MPP	1
23	Social worker	MSW	1
24	Nonprofit leadership	MA	1
25	Nonprofit leadership	BA	3
26	Nonprofit leadership	BA	3
27	Lawyer	JD	3
28	Nurse	RN	1
29	Social worker	MSW, PPSC	1
30	Social worker	BA	1
31	Nonprofit leadership	BA	1
32	Lawyer	JD	2
33	Lawyer	JD	3
34	Lawyer	JD	1
35	Nonprofit leadership	MS, LMFT	3
36	Nonprofit leadership	MS	1
37	Social worker	BA	1
38	Lawyer	JD	2
39	Lawyer	JD	1
40	Social worker	MSW, MA	1
41	Legal support staff	BA	3
42	Lawyer	JD	2

To begin the qualitative coding process, researchers developed a codebook as a team. Pursuant to Saldaña (2016), the codebook was reviewed periodically during team meetings as coding progressed to identify inconsistencies between coders, assess the codebook, and revise the coding scheme if changes were required (Saldaña, 2016). This iterative process resulted in a codebook that included a detailed description of each code, inclusion criteria for when to apply the code, exclusion criteria indicating when not to apply the code, and an example of excerpts included under the specific code. In total, researchers used 13 parent codes and 12 child codes. Two research team members led the coding process; each was arbitrarily assigned to code about half of the transcripts. They read the assigned transcripts and wrote first-impression analytic memos accessible to all team members. Subsequently, each transcript was coded using a series of first-cycle coding methods, including descriptive and process coding, as described in Saldaña (2016). These coding methods were used to identify an inventory of topics and common actions/processes raised and described by the participants. The coding process lasted 1 month.

After coding the transcripts, five research team members worked on writing analytical memos for each of the codes. The assignment of the codes was done arbitrarily. Team members organized and analyzed excerpts according to the participant group by writing analytic memos. Each memo included a summary of the Group 1, Group 2, and Group 3 perspectives and a section to compare and contrast the groups. This process aligns with the goals of second-cycle coding methods, specifically axial coding, given that it aimed to reorganize and reanalyze the data linking categories with subcategories (Saldaña, 2016). For example, a team member received the excerpts coded (during the first coding cycle) as part of the parent code “Shortfalls of the System” and applied axial coding to write an analytic memo summarizing each of the perspectives embedded in the specific parent code and comparing them. Thirteen analytic memos resulted from this analysis, and the seven with the richest data were prioritized. Analytic memo writing lasted approximately 1 month. Following the analytic memo writing process, the team met for a series of “Analysis of Preliminary Results Meetings,” where each member presented their analytic memo, and the group discussed how to conceptually group similar findings into results. All team members read all the analytic memos in preparation for these meetings.

## Results

Participants shared their perceptions and opinions on the intersections between intimate partner violence (IPV) and child welfare system (CWS) involvement. Most excerpts were coded into two of 13 different parent codes: Shortfalls of the System (289 excerpts) and Recommendations for Change (171 excerpts). The total excerpt count was 892. Three themes were identified from the participant’s responses. First, how IPV acts as an entry point to the CWS for children and their families. Second, the consequences related to CWS involvement for children and the parent-survivor in instances of IPV. Third, shortfalls of the CWS for both supporting families experiencing IPV and serving families more broadly were discussed in conjunction with recommendations for change. All names attributed to the excerpts are pseudonyms.

### Intimate Partner Violence as an Entry Point to the Child Welfare System

All participants agreed that IPV can be an entry point into the CWS. Participants commented on the multifactorial ways IPV serves as an entry point to the CWS via law enforcement involvement, mandated reporting, failure to protect charges alleged against the parent-survivor, and/or co-occurring factors associated with IPV, which can include substance use, poverty, race, and mental illness.

Several Group 1 professionals (child-focused social workers and lawyers) noted that most dependency cases involve IPV. Group 1 professionals acknowledged that fear of mandated reporting to child welfare officials can cause IPV survivors to forgo essential services. That resulting failure to leave the perpetrator may ultimately result in child removal. For example, Gemma, a Group 1 social worker, stated: “I feel like DV [domestic violence] is a huge part of many of our cases. There's what [a psychologist] used to call the trifecta, either mental health, substance abuse or domestic violence or any combination of those three issues.”

There's a separate count against mom for failure to protect. And so if she didn't leave right away, or she's covering up for the perpetrator when the social worker questions her, that's almost a guarantee that child be taken away from her too because that's an indication she's not protecting. (Rita, Social Worker, Group 1)

Group 2 (attorneys that represent parents) and Group 3 (leadership, attorneys, and social workers at DV shelters or IPV nonprofits) participants perceived CWS involvement secondary to IPV-related allegations of failure to protect to be unfair. Group 3 professionals, specifically, commented on how non-offending mothers, usually survivors of IPV, are at risk of losing custody of their children despite not being the perpetrator of violence.

…they [parent-survivors] are trying to walk through all of these decisions that they can make, but ultimately, when they do reach out for help, when they call the police, then you know, the possibility of their children being taken is very, very real. (Charlotte, Psychologist, Group 3)

### The Impact of Child Welfare System Involvement

When focusing on the impact of CWS involvement on families experiencing IPV, two main topics were discussed by participants, (1) harm inflicted on the child(ren) when family separation occurs and (2) consequences of CWS involvement for the parent-survivor.

Professionals from each group perceived family separation as a traumatic event for children that can have unintended consequences. For example, Rylie, a Group 1 social worker, said that “[w]hen they’re moved farther from their families and their communities, that is more trauma that is caused to the entire family, not to mention just the actual child. So there goes the depression issues [and] resistance to treatment.” Similarly, both Group 2 and Group 3 professionals contended that when removals occur due to IPV in the home, some “children are re-traumatized” (Raven, Attorney, Parent-oriented).

Participants from all three groups considered the termination of a parent’s custody of their child(ren) to be the most significant consequence of CWS involvement for parent-survivors. In addition, they also suggested that navigating the reunification process can be a significant challenge for parent-survivors. Participants suggested that parent-survivors must deal with the trauma of an abusive relationship while proving their ability to parent through the limited avenues of parental competence acknowledged by the CWS and the court.

Some of these people are married, you know, so now you're putting the additional burden of maybe having to get divorced, and the cost of getting divorced or just how difficult that actually is to do by yourself right, we're putting all of the onus on the victim to completely destroy their family, and just sort of putting somebody out in the cold and saying, ‘This is what keeps kids safe.’ (Courtney, Attorney, Group 1)

We see a lot of children being removed from victims of domestic violence, and why they have an allegation of failure to protect is because they're, they really have a short period of time to become engaged in services and to find alternative places to stay, pursue a restraining order. I mean, it's very difficult. (Jessica, Attorney, Group 2)

So all of those acts, [and] everything that the victim is doing to protect the family needs to be appreciated and reflected by the dependency court. Like okay, yes, there were times when you couldn't pacify your batterer, but you did your darndest to, to do that, so much of the time. (Regina, Attorney, Group 3)

Professionals from all three groups stated that CWS involvement may complicate parent-survivors’ lives without providing a clear benefit. This is perceived to be particularly salient when the parent-survivor and their family would likely benefit from supportive services that address the root causes of IPV rather than the imposition of non-tailored, mandatory interventions. Oftentimes, when the parent-survivor fails to comply with the mandatory interventions that a court imposes, the result is the removal of a parent-survivor’s child(ren) from their care.

The system forcing people to get services, which is so problematic in domestic violence cases, because here you have someone who's in a coercive relationship where someone's constantly telling them what to do. And then this other system comes into play and just replicates that power dynamic. And so that's also really problematic. (Manisha, Attorney, Group 2)

… [CWS involvement] it's just causing, it just causes so much harm to everyone in the family (…) it just seems like it would be so much better to be having them [parents], like, get into weekly therapy. Okay, you're not ready yet. Like, let's do a voluntary placement, let's track what's going on. Let me help you track what violent incidents have happened. (Regina, Attorney, Group 3)

Only a few participants spoke about the potential benefits of removal or positive effects of CWS involvement. One of these examples referred to a specific situation where a Group 1 social worker believed removal was warranted as the child thanked the social worker afterward: “I remember one particular student that was removed, temporarily and she was actually very thankful (…) she would come and tell me ‘thank you so much.’” (Kesha). In this example, it is suggested that given the severity of the violence happening in the household removal was warranted and offered a potential benefit for the child(ren). In another case, a Group 3 participant noted that “some children do thrive in their placements and they do really well, they have a good time.” (Anna, Attorney, Group 3).

### Shortfalls of the System and Recommendations for Change

Having worked in child welfare, specifically with families that experienced domestic violence, and they're the cases that nobody wants to be on. It's like the case that when you get a new case, and you see that it's domestic violence-related, sometimes, like your stomach drops, because they're really hard hard cases. They're very complex. (Penny, Researcher, Group 1)

#### Shortfalls of the System

Despite the overwhelming presence of IPV among families involved with the CWS, there was agreement among professional groups that the CWS was insufficient to address the complexity of IPV and child protection. Manisha, a Group 2 attorney, mentioned, “I have to say I never saw the child welfare intervention as helpful in any of the cases.” Similarly, Group 1 and Group 3 participants said:

The struggle with the department [DCFS] that we have with domestic violence, I think there's still, there's so much complexity to a family that's experiencing domestic violence that I think at times, it overwhelms our workers (...), and I think that leads us to have the same kind of set of treatment plans for all families as if they were universal, and that they were all experienced, seen it as the same, which the reality is all our families are uniquely different. (Danica, Social Worker, Group 1)

I’m honestly so sick of people telling me about trauma-informed care. And yet, we don't have trauma-informed systems. We don't have trauma-informed systems. If we had trauma-informed systems, we would be weighing up the harm, harm of staying in the home, harm of foster care, because at the end of the day, that's what we're weighing up, we're weighing up harm. (Kate, Nonprofit Leadership, Group 3)

More specifically, there was consensus across the three groups that harm is caused when child welfare investigations are conducted by those who do not have expertise in the field of IPV and/or have to make rapid decisions due to a heavy caseload. Participants believed that investigations conducted by social workers and DCFS employees without expertise may fail to account for cultural differences between the investigator and the family (which can lead to inaccurate evaluations of safety due to racism) and dynamics of power and control that can lead to inconsistent outcomes. “The system doesn't treat everyone the same way. But the reaction to domestic violence by DCFS just varies wildly. I would say it varies wildly by a social worker” (Martha, Social Worker, Group 1).

I think the social workers, you know, everybody beats up on the social workers, but they're not educated either (...) they haven't had the proper training. And I think that if they did have the proper training, they might handle it a little bit different[ly]. (Raven, Attorney, Group 2)

There continues to be a lack of information on the part of the social worker. Because again, you see these huge disparities where you, you might actually be wanting the department [DCFS] to remove children from the home children that you're you're working with? And they're like, no, there's no indices there, or I'm not going to do that. And then you see other children removed from the home where you're thinking, why on earth did you do that? (Kate, Nonprofit Leadership, Group 3)

As wonderful as DCFS social workers are you guys are way overburdened, and you cannot be that person, they need to have a professional working with them [survivors]. And there aren't enough of, there aren't enough professionals to help people. (Regina, Attorney, Group 3)

Some Group 2 and Group 3 professionals also stated that social workers have substantial latitude when conducting their investigation and deciding what or if intervention is necessary. It was suggested that individual discretion and lack of oversight can contribute to the variability in removals described by multiple participants, as well as allow for personal biases to influence these decisions.

One person shouldn’t have that much power um, to determine whether to remove children from a parent and that there was so much bias…it was just kind of luck of the draw if you've got somebody who was empathetic and tried to help and someone who was not. (Manisha, Attorney, Group 2)

Additionally, Group 2 and Group 3 professionals stated that harm is caused to survivors by other professionals in the court, particularly when interacting with judges who are not aware of or interested in the specific challenges that arise in abusive relationships. Jessica, a Group 2 attorney, stated: “One of the judges would, they'll scream and yell. I have people who've experienced trauma and it's infuriating and it's wrong…we have a lot of aggressors on the bench as well.”

We have some judges who are not as interested in the victim's point of view. And they find the fault or rather than, you know, seeing the full situation for what it is. And then they say, well, you're both [parent-survivor and aggressor] at fault.” (Anna, Attorney, Group 3)

At least one professional from each group made note of the racial disparities seen in the child welfare system; however, these observations were primarily made by the Group 2 and Group 3 professionals. “I think that people of color are disproportionately impacted, and there's racism in our system. And it strikes you when you walk in, and you see who's in the court system, it's not white people” (Jessica, Attorney, Group 2).

If you have a Native family who is requesting that cultural support, and traditional support, which they got on their reservations or (...) in the state, they can complete those programs through the tribes but the county and the courts do not see those services as appropriate. So basically, they don't count. So what happens is the family has to go through extra hoops in order to fulfill the department's idea of a successful program. (Rylie, Social Worker, Group 1)

The main thing that I hear over and over again, is that these, both, [systems that respond to] domestic violence and foster care (...) were created within the dominant culture. So, because of that, they are inherently problematic (...) based on a white supremacist culture rather than being inclusive. (Cameron, Program Manager, Group 3)

Moreover, one Group 2 participant argued that the state’s response to IPV is only punitive, despite the stated goals of the CWS.

We've all taken for granted, at least since the 70s, that these responses: mandatory arrest, orders of protection, jail, batters programs, child removal, foster care, anger management, that those are the responses that make sense. And I think it's because at the end of the day if we really sort of uncover what we're doing, it's a punitive response. It's not a response that's truly therapeutic, as the child protective court claims to be right. (Emily, Attorney, Group 2)

This participant suggested that the reasons for CWS intervention are often a result of systemic, societal injustices and that the CWS lacks the tools to appropriately change these societal ills and as a result is unable to meaningfully intervene in a way that is not punitive.

We have to stop responding to family poverty, which is, which is often at the root of all these things. The reason why it's not just some isolated incident is because it is true that we've been a country for a long time that has not done anything about structural racism. We're a country that hasn't done anything about resource hoarding…I get it why DCFS and ACS [Administration for Children’s Services] and CPS [Child Protective Services] are frustrated because they don't have the tools to address what these families are forced to experience in this country. (Emily, Attorney, Group 2)

#### Recommendations for Change

Participants from all groups agreed that change to the CWS was needed to improve outcomes for the families who become involved due to IPV. These changes primarily focused on using a more holistic approach to IPV intervention by engaging with the needs of families as a whole. However, some of the specific recommendations varied based on each group’s professional duties.

Professionals in Group 1, whose primary professional duty is to work in the child’s best interest, were more varied in their recommendations and were more likely to have conflicting perspectives. Some Group 1 professionals highlighted better training and collaboration with IPV experts as solutions for the previously described issues within the CWS. These participants also stated that they would like to offer preventative, continuous services, and individualized responses to IPV that focused on addressing the specific needs of the parent-survivor. For example, Danica said, “…help us make that assessment versus putting the pressure on our social workers to have to understand everything about substance abuse, everything about domestic violence, everything about mental health and all this stuff” (Social Worker, Group 1) and Rylie mentioned “When you're in a crisis, there's all of these services that can help you…So what about the prevention and education piece (…) what about the maintenance afterward” (Social Worker, Group 1).

I think we need to just take a step back and take a more nuanced approach to it. We need to think about the harm that investigations cause, we need to think about the harm even temporary removals cause…look a little more about whether we can provide reasonable efforts upfront and what those reasonable efforts can be. (Shannon, Directing Attorney, Group 1)

Other Group 1 professionals wanted to see large-scale, structural changes to address what they consider to be the root causes of IPV. Both Group 2 and Group 3 professionals, which included attorneys representing parents and people who worked at IPV nonprofits respectively, also suggested large-scale, structural changes. The recommended structural changes included providing direct support to families without child welfare involvement, specifically reallocating resources for children in the foster care system to the families and communities from which they are removed.

The resources that are going into the foster care system should be going into the community. And before people get into it, and not through the foster care system. (...) like CPS [Child Protective Services], DCFS partnering with shelters, that's, why not just give the shelters the money? Why not take the money away from DCFS? (Manisha, Attorney, Group 2)

It's been really heartbreaking for me to see this intersection [between IPV and child welfare system involvement in the family], because I think it's so avoidable if we can empower the victimized parent, whether it be mom or dad, to be able to leave financially, emotionally, if we can give them the resources. (Regina, Attorney, Group 3)

Additionally, many Group 2 professionals suggested that the entire CWS approach to IPV needs to be reimagined, including the suggestion to raise the foster care placement threshold to avoid the trauma of family separation. “Can we tolerate a little bit more just like, [the] tension in these families in order to prevent a child having more struggles by being removed?” (Allison, Attorney, Group 2).

You can't fix this approach, we need an entirely new response (...) that is not imagined by people for whom the system would never be right. Like, so I feel a lot of times this system was built by people who never expected it to come to their house. Because if they did, this would not be the system they built. (Emily, Attorney, Group 2)

Similarly, Group 3 professionals recommended more substantial changes, as compared to the more conservative recommendations from some Group 1 professionals. Many Group 3 professionals recommended centering the needs and lived experience of the parent-survivor, as well as abolishing the existing CWS. Anna, a Group 3 attorney, said “taking a look at the structure of the dependency system. (…) it's pretty racist and classist, and I think to really rip those out, you would have to take a look at the structure, in and of itself.”

I feel like there needs to be an entirely different way to deal with concerns about child safety. I'm more in the camp of like, getting rid of it [CWS] at this point (...) [it is] hard to accept that this thing actually isn't keeping kids safe, and it's actually causing more harm. And so then, you know, what else could we do that would be supportive. That's kind of where I'm at, unless, like, maybe not, maybe removing kids is like, really, somehow really restricted. Like, if we could (…) try to do other types of supportive work. (Elise, Attorney, Group 3)

What needs to change has to happen at the legislative level (...), the blind removal, you know, that showed us that if you take away bias, if you take away someone's ability to make a decision based on color, or ethnicity, or, or, or demographic (...) then decisions are made, just on the issues at hand, right? (...) then you don't find a system that's overrepresented by African American children and children of color, you don't find the inequality that happens when this system goes in to remove a child whose parent just simply called for help. I think what needs to change is, is the system is this whole system, and we know that it has to start at a much higher level than where we are. (Charlotte, IPV Nonprofit Leader, Group 3)

## Discussion

The majority of participants in this research study, no matter their professional background, readily acknowledged the harm that both removal and witnessing IPV cause children. This aligns with previous literature that has discussed the harm to children caused by both child removal and witnessing intimate partner violence (Danese & McEwen, 2012; Font & Gershoff, 2020; Mallett & Schall, 2019; Raz & Sankaran, 2019). Additionally, study aligns with previous research that shows that child welfare social workers and minors’ counsel (Group 1 professionals) seek to prioritize the well-being of children in their work pursuant to their professional and ethical duties (Berrick, 2019). However, among social workers associated with children’s legal services and minor’s counsel, there was a significant disconnect between an acknowledgment of harm and changing the way they perform their daily job duties to mitigate that harm. While Group 1 professionals offered recommendations to better support and serve families experiencing IPV, there was no discussion of efforts to implement these changes in their practice. On the other hand, parents’ attorneys and IPV nonprofit leadership and staff—Group 2 and Group 3 professionals respectively—talked about their everyday efforts to address the shortcomings of the existing CWS.

Several factors may contribute to the different approaches professionals take in addressing the child welfare system’s involvement in IPV cases. Primarily, this difference can be attributed to each professional’s role within the system. Each profession is constrained by its professional and legal obligations. For example, the job of parents’ attorneys (Group 2 professionals) and IPV nonprofit leadership and staff (Group 3 professionals) is to advocate for their parent-clients, which often involves pressing Group 1 professionals to question standard practice and tailor their decisions to each family’s needs and complex circumstances.

**Chart 1:**
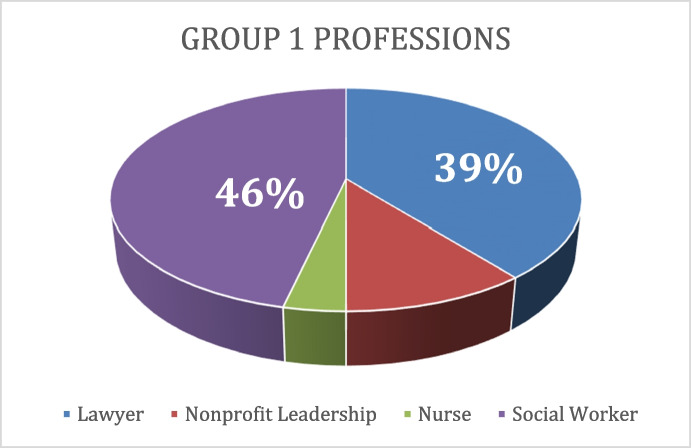
Group 1 professions

For child welfare social workers and minors’ counsel (Group 1 professionals), the data revealed additional factors contributing to their continued reliance on practices, such as child removal, in which they have expressed doubt. These additional factors include a generalized lack of training on IPV, obligations as mandated reporters, and large caseloads. Social workers, who made up the majority of the Group 1 professionals interviewed for the study, have been previously identified in research to perceive they lack IPV training and therefore lack the knowledge to address IPV with clients (Connor et al., 2012) (see Chart 1). This shortfall results in some Group 1 professionals feeling unprepared to support families experiencing situations of IPV, particularly using new or innovative methods beyond current CW practices. In contrast to Group 1 professionals, Group 2 and Group 3 professionals are often not mandated reporters and have increased training on the nuances of IPV.

It is also possible that mediatic pressure to take a risk-averse approach when intervening in cases of IPV contributes to the disconnect between acknowledging the shortcomings of the CWS and changing professional practices. Group 1 professionals may rely on foster care placement as a result of surveillance by both local and international media, which covers the failures of the CWS with great scrutiny, and the desire of CWS leadership to avoid this criticism (Cooper, 2005). The Netflix miniseries *The Trials of Gabriel Fernandez* is an example of this; it displays the case of an 8-year-old boy murdered by his mother and stepfather in Los Angeles County and Los Angeles County’s Department of Children and Family Services (DCFS) handling of the case. *The Trials of Gabriel Fernandez* highlights the duty and power social workers have in protecting children by interviewing the social workers and supervisors who were charged with and acquitted of criminal liability in Gabriel’s case (Hinkamp, 2021).

Among Group 1 professionals, there was some disagreement as to the recommendations for reforming the CWS to better support families. One Group 1 professional saw removal as a necessary and appropriate response to IPV in most cases and thought that current systems did not need to be completely upended. However, many other Group 1 professionals stated that there should be more effort to understand IPV and prevent child removal through resources, support, training, and changes to the system.

On the other hand, there was more consensus among Group 2 and Group 3 professionals on recommendations for changing the child welfare involvement of families experiencing IPV. Group 2 and Group 3 professionals focused on systemic problems in the CWS, highlighting the existing racial disproportionality, and therefore called for a more complete system overhaul (Dettlaff & Boyd, 2020). These parents’ attorneys and IPV nonprofit leadership and staff felt frustrated by consistent calls for training. They viewed training as helpful and needed but felt that past efforts to train CWS professionals did not lead to necessary change. Instead, many Group 2 and Group 3 professionals suggested more radical changes that addressed the root causes of IPV and CWS involvement. These suggestions ranged from calls to abolish the CWS as it currently exists, reform or rescind mandated reporting laws, and learning from and employing traditional cultural approaches to IPV intervention and family support. Leadership and staff affiliated with IPV nonprofits emphasized the need for tailored family services that are culturally specific and racially appropriate.

Interestingly, most participants focused on the shortfalls of the current CWS and the harm of family separation; discussions did not center on the harm caused by witnessing IPV during childhood. One possible explanation for this finding is that participants may have felt that the harm caused by witnessing IPV was self-explanatory and chose to use these interviews as an opportunity to highlight the shortfalls of our current CWS response to these issues. Since the discussions primarily focused on the harm caused by CWS involvement, these professionals may view the harm of family separation as greater than the harm of IPV exposure, particularly in light of the recent advocacy efforts that have highlighted racial disparities in the CWS. Work on racial disparities in the CWS has demonstrated how damaging the CWS has been historically when intervening into the lives of families of color and how this harm continues today (Turner Hogan & Siu, 1988; Chasnoff et al., 1990; Lane et al., 2002; Rivaux et al., 2008; Merritt, 2021).

The literature is unclear about whether removal is an appropriate response to children witnessing IPV. In cases of child abuse, the harm is well-documented and the benefits of foster care placement are understood (Font & Gershoff, 2020). However, *witnessing* IPV may be different than what is commonly considered to be child *abuse*. Research is not clear on the benefits of family separation in cases where *witnessing* IPV is charged as child neglect, particularly when considering the harm that a child may encounter during family separation and foster care placement (Font & Gershoff, 2020). Group 2 and Group 3 participants seemed reluctant to endorse the intervention of the CWS in cases of neglect derived from *witnessing* IPV as this practice fails to acknowledge efforts made by parent-survivors to protect and care for their child(ren), and punishes them for their victimization.

### Strengths and Limitations

Some limitations should be kept in mind when interpreting these results. Because secondary data was used in this study, focus group discussions and interview protocols were not specifically aimed at answering our research questions. For example, participants were not explicitly asked how their professional orientation shapes their perception of the harm caused by witnessing IPV during childhood versus the harm that may be caused by removal from the household. Therefore, this opens a door for future research to directly design and implement data collection strategies to identify professionals’ strategies to balance the harm of witnessing IPV versus removal. In addition, the data collection protocols primed participants to think of IPV as a perpetrator-against-victim situation excluding narratives of mutual violence between partners. Thus, future research should act upon this limitation. Furthermore, there was likely self-selection among professionals who participated in this study. Those who responded to the recruitment of the original study were likely skewed towards those who were more likely to criticize the status quo.

While this is not a limitation, the context in which these interviews were conducted should be acknowledged. The interviews were conducted between August and December 2020. At that time, the COVID-19 pandemic was still devastating communities, and a COVID-19 vaccine was not FDA-approved. Furthermore, interviews occurred in the wake of significant national attention towards systemic racism in all aspects of American society, derived from a civil uprising and the Black Lives Matter movement following the murder of George Floyd.

One strength of this study is the rich rigor of the collected data, as defined by Tracy (2010). The current study used abundant data and reached thematic saturation substantiating results and claims. Moreover, it involved collaborative analysis processes by a multidisciplinary team of researchers from the disciplines of medicine, law, and social science, engaging multiple analytic viewpoints throughout, resulting in a rich analysis that brings together views from different individuals and disciplines.

### Implications for Practice

Based on the results of this research, it is clear that Los Angeles County’s CWS must be significantly reformed with regard to how it intervenes in cases of IPV. Many advocates and policymakers have recently proposed bringing together multidisciplinary teams to put forward policy changes, make child removal decisions, and evaluate child removal decisions (Bai et al., 2019; Bath et al., 2020; Herbert & Bromfield, 2019; Ogbonnaya & Keeney, 2018; Trubek & Farnham, 2000). Our study’s findings illuminate how each professional’s professional and legal duties influence their decision-making within the CWS. Because these professionals make decisions that significantly impact the lives of families, a multidisciplinary approach to policy creation and CWS decision-making is necessary. Increased collaboration between representatives of children, parent-perpetrators, and parent-survivors would allow for a more accurate assessment of the harms and benefits of CWS involvement for all family members impacted by IPV. A multidisciplinary team may reduce the variability in CWS response, ensuring families receive the care and services that are most appropriate to their specific needs.

While improved training on IPV and related issues may be beneficial, our findings suggest it is not sufficient to address the current shortfalls of the CWS. Policies should be altered to allow CWS professionals to intervene in homes where IPV is occurring by centering on the needs of the parent-survivor. Support should be provided that allows the parent-survivor to maintain custody of and develop protective, secure attachments with their child(ren) as they navigate the challenges of their abuse. In doing so, we will enhance the well-being of the whole family, including the child(ren) who will be protected from possible harms of removal and foster care placement. Although each member of the family may have competing needs and interests, a plan can be made to balance all of these interests and prioritize the safety of each family member without resorting to tearing the family apart. There is a way for the family to coexist healthily if given adequate support that focuses on preventing IPV and child abuse. Future research will hopefully reveal what prevention mechanisms are necessary both in the structure of the child welfare system and in the way families are evaluated by the CWS.

## Conclusion

To our knowledge, this is the first qualitative study to investigate the role that professional orientation plays in assessing the harm of foster care placement in the context of IPV. The results of this study strongly suggest that reform is needed, and further research is needed to better understand what changes would best serve the diverse needs of families struggling with IPV and resulting CWS involvement.

## Data Availability

The dataset analyzed in the study is not publicly available due to the UCLA Pritzker Center for Strengthening Children and Families being the entity with the right to share. However, partial data may be available from the corresponding author after approval from the UCLA Pritzker Center for Strengthening Children and Families upon reasonable request.
